# Epidemiology and outcomes of *Candida*-associated osteoarticular infections: A multicentre retrospective study from Turkey

**DOI:** 10.1093/mmy/myaf080

**Published:** 2025-09-01

**Authors:** Özlem Güler, Murat Üzel, Dilşat Tepe, Firdevs Aksoy, Güle Çınar, Kemal Osman Memikoğlu, Bülent Durdu, Tülay Ünver Ulusoy, Aysun Benli̇, Zeynep Karakaşoğlu, Neşe Saltoğlu, Derya Seyman, Işıl Deniz Alıravcı, Hasibullah Yaqoobi, Ayça Aydın, Hatun Öztürk Çerik, Selin Özdemir, Betül Sümer, Azize Yetişgen, Ayşe Batırel, Ayşe Serra Özel, Seniha Şenbayrak, Yasemin Tezer Tekçe, Zehra Çağla Karakoç

**Affiliations:** Department of Infectious Diseases and Clinical Microbiology, Kocaeli University, Faculty of Medicine, Kocaeli 41380, Turkey; Department of Orthopaedics and Traumatology, Hand Surgery, Kocaeli University, Faculty of Medicine, Kocaeli 41380, Turkey; Department of Infectious Diseases and Clinical Microbiology, Karadeniz Technical University, Faculty of Medicine, Trabzon 61080, Turkey; Department of Infectious Diseases and Clinical Microbiology, Karadeniz Technical University, Faculty of Medicine, Trabzon 61080, Turkey; Department of Infectious Diseases and Clinical Microbiology, Ankara University, Faculty of Medicine, Ankara 06230, Turkey; Department of Infectious Diseases and Clinical Microbiology, Ankara University, Faculty of Medicine, Ankara 06230, Turkey; Department of Infectious Diseases and Clinical Microbiology, Bezmialem Vakif University, Faculty of Medicine, Istanbul 34093, Turkey; Department of Infectious Diseases and Clinical Microbiology, Ankara Etlik City Hospital, Ankara 06170, Turkey; Department of Infectious Diseases and Clinical Microbiology, Istanbul University, Istanbul Faculty of Medicine, Istanbul 34093 , Turkey; Department of Infectious Diseases and Clinical Microbiology, Istanbul University-Cerrahpaşa, Cerrahpaşa Faculty of Medicine, Istanbul 34098, Turkey; Department of Infectious Diseases and Clinical Microbiology, Istanbul University-Cerrahpaşa, Cerrahpaşa Faculty of Medicine, Istanbul 34098, Turkey; Department of Infectious Diseases and Clinical Microbiology, Health Sciences University, Antalya Education and Research Hospital, Antalya 07100, Turkey; Department of Infectious Diseases and Clinical Microbiology, Çanakkale 18 Mart University, Faculty of Medicine, Çanakkale 17020, Turkey; Department of Infectious Diseases and Clinical Microbiology, Başkent University, Faculty of Medicine, Adana Application and Research Centre Yüreğir Hospital, Adana 01250, Turkey; Department of Infectious Diseases and Clinical Microbiology, Sultan 2. Abdulhamid Han Education and Research Hospital, Istanbul 34668, Turkey; Department of Infectious Diseases and Clinical Microbiology, Ordu University, Faculty of Medicine, Ordu 52200, Turkey; Department of Infectious Diseases and Clinical Microbiology, Health Sciences University, Izmir Bozyaka Education and Research Hospital, Izmir 35170, Turkey; Department of Infectious Diseases and Clinical Microbiology, Erzincan Binali Yıldırım University, Mengücek Gazi Education and Research Hospital, Erzincan 24100, Turkey; Department of Infectious Diseases and Clinical Microbiology, Turgut Özal University, Malatya Education and Research Hospital, Malatya 44000, Turkey; Department of Infectious Diseases and Clinical Microbiology, Health Sciences University, Kartal Dr. Lütfi Kırdar City Hospital, Istanbul 34865, Turkey; Department of Infectious Diseases and Clinical Microbiology, Health Sciences University, Ümraniye Education and Research Hospital, Istanbul 34764, Turkey; Department of Infectious Diseases and Clinical Microbiology, Health Sciences University, Haydarpaşa Numune Education and Research Hospital, Istanbul 34668, Turkey; Department of Infectious Diseases and Clinical Microbiology, Health Sciences University, Ankara City Hospital, Ankara 06800, Turkey; Department of Infectious Diseases and Clinical Microbiology, Istınye University, Faculty of Medicine, Istanbul 34010, Turkey

**Keywords:** *Candida*, arthritis, bone diseases, infectious, osteomyelitis, spondylodiscitis

## Abstract

This multicentre retrospective study investigated the epidemiology, clinical characteristics, and fluconazole resistance rates of *Candida* species in osteoarticular infections across Turkey as well as the factors influencing complete recovery. Data were gathered from 73 adult patients diagnosed with proven or probable *Candida*-associated osteoarticular infections between 2015 and 2025 from 20 healthcare centres. The most common clinical presentation was spondylodiscitis, followed by the involvement of phalangeal bones in the hands and feet. Non-*albicansCandida* species accounted for 37/73 cases (50.7%), with *Candida parapsilosis* being the most frequent. Fluconazole resistance was low among *C. albicans* isolates (3%) but higher among non-*albicans* yeasts (27%). Bacterial co-infection, predominantly Gram-positive bacteria, was detected in 52.1% of cases. Diabetes was present in 50/73 patients (68.5%), particularly insulin-dependent diabetes, and was a prominent comorbidity that may have also contributed as a predisposing factor. Radiological detection of osteomyelitis was achieved in 69.9% of patients. Fluconazole was the most commonly used antifungal agent (74%) with a median treatment duration of 90 days. Multivariate analysis revealed that surgical debridement was significantly associated with a higher odds of clinical recovery (adjusted odds ratio [aOR], 5.764; 95% confidence interval [CI], 1.360–24.434; *P* = .017), whereas diabetes mellitus was significantly associated with a lower odds of total recovery (aOR, 0.205; 95% CI, 0.053–0.792; *P* = .022). In conclusion, this multicentre study provides epidemiological data and fluconazole resistance rates of *Candida* species causing osteoarticular infections in Turkey, highlights the occurrence of *C. auris* in this cohort, and identifies surgical intervention and diabetes mellitus as factors significantly associated with recovery.

## Introduction

COVID-19/fungal co-infections, including those caused by *Candida* species, gained increased attention during the pandemic. In 2022, the World Health Organization (WHO) published its first fungal priority pathogens list, which included *Candida albicans* and *C. auris* in the ‘critical priority’ group.^1,2^*Candida* species can cause diverse clinical manifestations, ranging from cutaneous to mucosal and systemic infections, including candidaemia.^3^

Candidal osteoarticular infections are known to develop more frequently after candidaemia but are quite rare, with an incidence of 0.4% among adult candidaemia cases.^4,5^ Diagnosis is often challenging, and management is particularly difficult, as these infections may require aggressive surgical intervention in some cases, in addition to prolonged antifungal treatment.^6^

The objective of this study is to investigate the epidemiology and fluconazole resistance rates of *Candida* species in osteoarticular infections across all accessible centres in Turkey. In addition, the demographic and clinical characteristics of the affected patients will be evaluated. Furthermore, this study aims to assess the role of newly emerging fungal pathogens in these rare infections, such as *C. auris*, which has been classified as a critical priority pathogen since its emergence in 2009, possibly due to climate change.^1^ Finally, the factors influencing clinical improvement in patients with osteoarticular *Candida* infections will be investigated.

### Patients and methods

This retrospective cohort study was supported by the Turkish Society of Clinical Microbiology and Infectious Diseases. Patients diagnosed with proven and probable osteoarticular infections caused by *Candida* spp. in various healthcare facilities in Turkey between 2015 and 2025 were included in this study.^6^ Osteoarticular infections due to *Candida* spp. are rare; therefore, all proven and probable patients were included in the study. As paediatric and adult infectious diseases are separate departments, and the ethics committee did not approve the inclusion of paediatric patients, they were excluded from the study.

Records of adult patients with osteomyelitis, arthritis, and spondylodiscitis from a range of socioeconomic backgrounds were reviewed. In patients with clinical and radiological findings suggestive of osteoarticular infection, a diagnosis of proven *Candida*-associated osteoarticular infection is made if *Candida* spp. were isolated from the bone tissue, synovial fluid, or metal prosthesis components. Conversely, when *Candida* spp. were isolated from other adjacent samples, such as pus, disc, cartilage, tendon, and adjacent abscess, it is defined as probable *Candida*-associated osteoarticular infection.^6^ Patients with *Candida* growth on the skin were excluded from the study to rule out the possibility of contamination or colonisation. Depending on the resources of the health centre, yeast identification was performed using one of the following methods: matrix-assisted laser desorption/ionisation time-of-flight mass spectrometry, the VITEK® 2 automated system (bioMérieux, France), or the Phoenix automated system (Becton, Dickinson and Company, Franklin Lakes, NJ, USA). Antifungal susceptibility testing was primarily performed using the VITEK® 2 system (bioMérieux, France), which was the most widely used method across participating centres. In addition, some centres utilised alternative methods such as the Sensititre YeastOne colorimetric microdilution system (Thermo Fisher Scientific, France), the Micronaut-AM microdilution system (MERLIN Diagnostika GmbH, Germany), and gradient diffusion methods (e.g., Etest) for antifungal resistance testing. These tests were interpreted in accordance with the guidelines established by the European Committee on Antimicrobial Susceptibility Testing and recommendations of the Turkish Ministry of Health. Isolates were considered resistant when the minimum inhibitory concentration threshold value was greater than 16 mg/l for *C. glabrata* and greater than 4 mg/l for other *Candida* species. *Candida krusei* was excluded from the evaluation because of its intrinsic resistance.^7^

The following demographic data were collected: age, sex, city of residence, rural residence, and comorbid diseases, such as diabetes mellitus, insulin treatment, chronic renal failure, dialysis, chronic liver disease, cirrhosis, chronic lung disease, haematological malignancy, solid organ malignancy, haematogenous stem cell transplantation, solid organ transplantation, HIV or SARS-CoV-2 infection, hypogammaglobulinemia, rheumatoid arthritis, and gouty arthritis. The following factors were considered predisposing factors for osteoarticular *Candida* spp. infection and were recorded: the use of steroids or other immunosuppressive agents; the presence of a central venous catheter; total parenteral nutrition; a history of major surgical intervention in the head, neck, thoracic, or abdominal regions within the last month; the use of broad-spectrum antibiotics; a history of trauma to the affected joint or bone; injection of steroids or other drugs into the affected joint; and the presence of a joint prosthesis.^6,8^

A comprehensive evaluation of the patients’ symptoms and examination findings included the presence of pain, restriction in joint movements, localised oedema and warmth, fistula orifice with drainage, systemic fever, and neurological deficits. The duration from the onset of symptoms to diagnosis was documented in days. At the time of diagnosis, the following biochemical parameters were recorded: serum glucose (mg/dl), counts for leukocyte (µl), neutrophil (µl), lymphocyte (µl), concentration of C-reactive protein [CRP] (mg/l), erythrocyte sedimentation rate (mm/h), blood urea nitrogen (mg/dl), creatinine (mg/dl), and albumin (g/dl) levels.

A detailed examination was conducted to investigate the presence of candidaemia, endocarditis, *Candida* retinitis, and candiduria. The presence of concurrent bacterial growth and antifungal resistance patterns in *Candida* spp. was determined.

In the context of radiological evaluation, imaging modalities available to patients were documented, including direct radiographs, computed tomography (CT), and magnetic resonance imaging (MRI). The presence of fracture, osteolysis, osteomyelitis, periosteal reaction, bone abscess, or sequestrum was assessed. The contribution of nuclear medicine to the diagnostic process was also evaluated.

Regarding the treatment process, the type and duration of antifungal therapy administered, whether surgical intervention was performed, and the total follow-up period of the patients were recorded.

Patients were analysed in two groups: those with complete recovery and those with partial recovery. Complete recovery of the patient was defined as the total resolution of both clinical and radiological findings. Partial recovery was defined as partial resolution of clinical and/or radiological findings or partial recovery in the absence of radiological data.^6^

Statistical analyses were performed using IBM SPSS, version 29.0 (IBM Corp., Armonk, NY, USA). The normality of continuous variables was assessed using the Kolmogorov–Smirnov test. Data following a normal distribution were expressed as mean ± standard deviation (SD), whereas data not following a normal distribution were presented as median and interquartile range. Categorical variables were reported as counts and percentages. The statistical significance of differences between groups was assessed using the independent *t*-test or Mann–Whitney *U* test, depending on the distribution of the data. The *Χ*^2^ test was used to analyse the relationships between categorical variables. To identify the risk factors associated with complete recovery, univariate logistic regression analyses were performed first, and significant variables were included in the multivariate logistic regression analysis. Results with a *P*-value < .05 were considered statistically significant.

This study was reported in accordance with the Strengthening the Reporting of Observational Studies in Epidemiology statement.^9^

## Results

This study included 73 patients diagnosed with osteoarticular infections caused by *Candida* spp. over 10-year period, based on data collected from 20 healthcare centres located across various provinces. All the participating hospitals were large multidisciplinary tertiary care centres, including university hospitals and major training and research hospitals. They serve patients with a broad range of socioeconomic backgrounds. The included hospitals cover six of the seven geographical regions of the country. The patients were residents of 21 cities, including Adana, Ankara, Antalya, Çanakkale, Çankırı, Çorum, Denizli, Diyarbakır, Erzincan, İstanbul, İzmir, Kırıkkale, Kocaeli, Kütahya, Malatya, Manisa, Mersin, Muş, Ordu, Sivas, and Trabzon.

The type of osteoarticular infection, demographic characteristics of the patients, underlying comorbidities, and predisposing factors for infection are summarised in Table 1. In addition to Table 1, other rare conditions in patients included the need for dialysis (*n* = 4), chronic liver disease (*n* = 4), haematogenous malignancy (*n* = 4), solid organ malignancy (*n* = 4), total parenteral nutrition (TPN; *n* = 3), cirrhosis (*n* = 2), intra-articular steroid injection (*n* = 2), intra-articular administration of a non-steroid drug (*n* = 2), haematopoietic stem cell transplantation (*n* = 1), solid organ transplantation (*n* = 1), hypogammaglobulinaemia (*n* = 1), and gouty arthritis (*n* = 1). None of the patients had a known HIV infection; all were tested and found to be negative. Recently, treatments for osteoarthritis have frequently included injection therapies such as intra-articular steroids and platelet-rich plasma.^10^ However, in the cohort of patients in our study, only two patients received intra-articular steroid injections, and two patients received various intra-articular pharmacological agents.

**Table 1.** Demographic characteristics in cases of *Ca ndida* osteoarticular infections.

Characteristic	Total cohort *n* = 73	Patients with partial recovery, *n* = 37	Patients with complete recovery, *n* = 36	*P*-value
Osteomyelitis, *n* (%)	56 (76.7)	29 (78.4)	27 (75)	.949^†^
Arthritis, *n* (%)	29 (39.7)	14 (37.8)	15 (41.7)	.924^†^
Spondylodiscitis, *n* (%)	15 (20.5)	6 (16.2)	9 (25)	.523^†^
Involvement of multiple bones, *n* (%)	26 (35.6)	15 (40.5)	11 (30.6)	.518^†^
Age in years, median (IQR)	65 (57.5–74)	67 (58–75)	63.5 (56.25–71)	.299^‡^
Sex, *n* (%)				.198^†^
Female	30 (41.1)	12 (32.4)	18 (50)	
Male	43 (58.9)	25 (67.6)	18 (50)	
Residence in a rural area, *n* (%)	20 (27.4)	11 (29.7)	9 (25)	.849^†^
Comorbidities, *n* (%)				
Diabetes mellitus	50 (68.5)	30 (81.1)	20 (55.6)	**.036^†^**
Requirement for insulin therapy	34 (46.6)	22 (59.5)	12 (33.3)	**.045** **^†^**
Chronic renal failure	20 (27.4)	11 (29.7)	9 (25)	.849**^†^**
Chronic lung disease	8 (11)	2 (5.4)	6 (16.7)	.152**^†^**
Rheumatoid arthritis	5 (6.8)	0 (0)	5 (13.9)	**.025** **^†^**
COVID-19	5 (6.89	2 (5.4)	3 (8.3)	.674**^†^**
Predisposing factors, *n* (%)				
Corticosteroid therapy	11 (15.1)	2 (5.4)	9 (25)	**.044** **^†^**
Other immunosuppressant therapy	11 (15.1)	4 (10.8)	7 (19.4)	.482**^†^**
Central venous catheter	13 (17.8)	6 (16.2)	7 (19.4)	.957**^†^**
Broad-spectrum antibiotic use in the last month	59 (80.8)	30 (81.1)	29 (80.6)	1**^†^**
Major surgery in the last month	45 (61.6)	21 (56.8)	24 (66.7)	.529^†^
Head and neck surgery	32 (43.8)	18 (48.6)	14 (38.9)	
Thoracic surgery	10 (13.7)	3 (8.1)	7 19.4)	
Abdominal surgery	3 (4.1)	0 (0)	3 (8.3)	
History of trauma or surgery to the affected joint or bone	35 (47.9)	17 (45.9)	18 (50)	.911^†^
Joint prosthesis	25 (34.2)	11 (29.7)	14 (38.9)	.563^†^

COVID-19, coronavirus disease 2019; SD, standard deviation; IQR, interquartile range.

† *Χ* ^2^ test.

‡ Mann–Whitney *U* test.

The identified *Candida* spp. and their fluconazole resistance profiles are presented in Table 2. *Candida auris* was isolated in only one of the 73 cases. The patient was a 41-year-old male who underwent surgery for multiple traumas and L1–L2 vertebral fractures following a fall from a height. The patient’s only comorbidity was insulin-dependent diabetes mellitus, a major risk factor for infection. During the postoperative period, deep tissue cultures obtained due to the development of pain, swelling, redness, increased discharge, and fistula formation at the wound site revealed the growth of *C. auris* and *Klebsiella pneumoniae*. The isolated *C. auris* was resistant to fluconazole, voriconazole, and amphotericin B, but susceptible to echinocandins. The patient received caspofungin therapy for 49 days. The infection was eradicated; however, the follow-up period, including treatment, was only 49 days at the time of the study; therefore, recurrence could not be excluded. The clinical and radiological findings were not completely resolved.

**Table 2.** Detected *Candida* species.

*Candida* species	*n* (%)	Fluconazole resistance, *n*
*Candida albicans*	36 (49.3)	1
*Candida parapsilosis*	15 (20.5)	5
*Candida glabrata*	7 (9.6)	0
*Candida tropicalis*	7 (9.6)	0
*Candida guilliermondii*	2 (2.7)	1
*Candida krusei*	2 (2.7)	2
*Candida auris*	1 (1.4)	1
*Candida kefyr*	1(1.4)	0
*Candida magnoliae*	1 (1.4)	1
*Candida melibiosica*	1 (1.4)	0
Total	73	11

Concurrent bacterial growth with *Candida* spp. was detected in 38 cases. Coagulase-negative *Staphylococcus* species were isolated in 23.6% of cases (*n* = 9), making this group the most frequently encountered bacteria. The second most common concurrent bacterial pathogen was *Enterococcus* spp., which were isolated from six patients (15.8%). *Staphylococcus aureus* and *K. pneumoniae* were detected in four cases (10.5%). In addition, *Pseudomonas aeruginosa* was identified in three cases, and *Streptococcus* spp. and *Enterobacter cloacae* in two cases. A particularly noteworthy finding was the simultaneous growth of *Salmonella typhimurium* in the tissue culture of one patient.

Table 3 outlines the anatomical locations of the osteoarticular infections. Spondylodiscitis was the most common clinical condition. This was followed by the involvement of phalangeal bones in the hands and feet. It is important to note that the involvement of the phalanges is almost always accompanied by the involvement of the interphalangeal joints. This leads to the involvement of multiple bones and significant functional impairment.

**Table 3.** Anatomical sites affected by *Candida* osteoarticular infections.

Affected bone or joint	*n* (%)
Vertebrae and intervertebral discs	15 (20.5)
Phalanx	12 (16.4)
Knee joint	11 (15.1)
Metatarsal bone	8 (11)
Sternum	7 (9.6)
Hip joint	6 (8.2)
Femur	4 (5.5)
Tibia	3 (4.1)
Temporomandibular joint and mastoid bone	2 (2.7)
Base of the skull	1 (1.4)
Humerus	1 (1.4)
Mandible and mastoid bone	1 (1.4)
Medial cuneiform bone	1 (1.4)
Temporal bone	1 (1.4)

The symptoms, findings, and results of the radiological imaging studies of osteoarticular *Candida* spp. infections are summarised in Table 4. The diagnostic process included bone scintigraphy in eight patients, labelled leukocyte scintigraphy in one patient, and positron emission tomography imaging in one patient. Radioactive tracer uptake consistent with infection was detected in eight patients who underwent nuclear imaging.

**Table 4.** Symptoms, clinical findings, and radiological imaging results of *Candida* osteoarticular infections.

Symptoms and findings	Total cohort*n* = 73	Patients with partial recovery	Patients with complete recovery	*P*-value
Duration from onset of symptoms to diagnosis in days, median (IQR)	30 (16.5–90)	30 (14.5–90)	30 (17.75–83)	.761^‡^
Pain, *n* (%)	70 (95.9)	36 (97.3)	34 (94.4)	.615^†^
Restriction in movements, *n* (%)	59 (80.8)	29 (78.4)	30 (83.3)	.810^†^
Oedema, *n* (%)	57 (78.1)	28 (75.7)	29 (80.6)	.825^†^
Localised warmth, *n* (%)	53 (72.6)	29 (78.4)	24 (66.7)	.390^†^
Fistula orifice with drainage, *n* (%)	27 (37)	16 (43.2)	11 (30.6)	.379^†^
Fever, *n* (%)	29 (39.7)	12 (32.4)	17 (47.2)	.293^†^
Neurological deficit, *n* (%)	13 (17.8)	10 (27)	3 (8.3)	.075^†^
Serum glucose (mg/dl), median (IQR)	118 (94–168)	127 (98–185)	110 (91–164)	.292^‡^
Total leucocytes (µl), mean ± SD	8901.04 ± 3779.96	8952.59 ± 3730.68	8848.06 ± 3882.17	.907 ^§^
Neutrophil (µl), median (IQR)	5240 (3850–7800)	5060 (3720–7330)	5515 (3905–8050)	.873^‡^
Lymphocyte (µl), mean ± SD	1687.05 ± 796.65	1641.22 ± 807.68	1734.17 ± 793.77	.622^§^
CRP (mg/l), median (IQR)	31 (5.5–80.5)	26 (6.5–73.5)	43.5 (5.25–87.25)	.558^‡^
Sedimentation rate (mm/h), mean ± SD	54.18 ± 28.7	52.22 ± 23.64	56.19 ± 33.34	.559 ^§^
Blood urea nitrogen (mg/dl), median (IQR)	27 (17–46)	30 (16.5–58.50)	24 (18–37.5)	.202^‡^
Creatinine (mg/dl), median (IQR)	0.89 (0.64–1.36)	0.9 (0.74–1.59)	0.82 (0.61–1.29)	.251^‡^
Albumin (g/l), mean ± SD	33.26 ± 7.15	32.81 ± 7.76	33.72 ± 6.54	.590 ^§^
Candidaemia, *n* (%)	5 (6.8)	2 (5.4)	3 (8.3)	.674^†^
Concomitant bacterial growth, *n* (%)	38 (52.1)	20 (54.1)	18 (50)	.911^†^
Type of radiological imaging, *n* (%)				
Direct radiography	57 (78.1)	28 (75.7)	29 (80.6)	.825^†^
Computed tomography	32 (43.8)	19 (51.4)	13 (36.1)	.282^†^
Magnetic resonance imaging	46 (63)	24 (64.9)	22 (61.1)	.929^†^
Radiological Finding, *n* (%)				
Fracture	8 (11)	5 (13.5)	3 (8.3)	.711^†^
Osteomyelitis on imaging	51 (69.9)	26 (70.3)	25 (69.4)	1^†^
Osteolysis	36 (49.3)	19 (51.4)	17 (47.2)	.906^†^
Periosteal reaction	20 (27.4)	8 (21.6)	12 (33.3)	.390^†^
Bone abscess	15 (20.5)	9 (24.3)	6 (16.7)	.603^†^
Sequestrum	9 (12.3)	7 (18.9)	2 (5.6)	.152^†^

SD, standard deviation; IQR, interquartile range.

† *Χ* ^2^ test.

‡ Mann–Whitney *U* test.

§ Independent *t*-test.

No concomitant endocarditis was found in any patient. Candiduria was observed in four patients, and *Candida* retinitis was diagnosed in one patient. Other clinical findings included paravertebral abscess in three patients and myositis in three patients. In addition, two patients presented with retropharyngeal abscesses, and one patient had a psoas abscess associated with spondylodiscitis.

Information on antifungal treatment and patient follow-up is presented in Table 5. In addition, one patient was treated with itraconazole, and the other with posaconazole. Four patients died during follow-up.

**Table 5.** Treatment and outcome of *Candida* osteoarticular infections.

Treatment and outcome	Total cohort*n* = 73	Patients with partial recovery	Patients with complete recovery	*P*-value
Fluconazole, *n* (%)	54 (74)	31 (83.8)	23 (63.9)	.095^†^
Voriconazole, *n* (%)	9 (12.3)	4 (10.8)	5 (13.9)	.736^†^
Anidulofungin, *n* (%)	6 (8.2)	3 (8.1)	3 (8.3)	1^†^
Caspofungin, *n* (%)	10 (13.7)	6 (16.2)	4 (11.1)	.736^†^
Micafungin, *n* (%)	13 (17.8)	3 (8.1)	10 (27.8)	.59^†^
Liposomal amphotericin B, *n* (%)	6 (8.2)	3 (8.1)	3 (8.3)	1^†^
Duration of treatment in days, median (IQR)	90 (38.5–129)	76 (28–111)	90 (43.5–180)	**.032** ^‡^
Duration of follow-up in days, median (IQR)	180 (57.5–360)	120 (44.5–360)	215 (66.5–365)	.213^‡^
Surgical treatment, *n* (%)	52 (71.2)	21 (56.8)	31 (86.1)	**.012** ^†^

SD, standard deviation; IQR, interquartile range.

† *Χ* ^2^ test.

‡ Mann–Whitney *U* test.

Among patients who did not undergo surgery, some died within a short period after diagnosis, some declined surgical intervention, in certain cases (e.g., post-sternotomy infections), the anatomical site was unsuitable for surgery, and in others, surgery was not performed because of high operative risk. Surgical procedures performed included abscess drainage, amputation, debridement, discectomy, mastoidectomy, and prosthesis removal. Multivariate analysis for factors influencing a complete cure was performed using the logistic regression method, and the results are shown in Table 6. The analysis showed that the presence of diabetes and surgical intervention during treatment were significantly associated with complete cure. Recovery was not statistically associated with fluconazole resistance or whether the infection was caused by *C. albicans* or non-*albicans* species.

**Table 6.** Multivariate analysis of factors affecting complete recovery using logistic regression.

	Univariate		Multivariate	
Variable	OR (95% CI)	*P*	aOR (95% CI)	*P*
Duration of treatment	1.003 (0.998–1.007)	.261	1.004 (0.998–1.011)	.154
Diabetes mellitus absent (R)	0.292 (0.102–0.836)	**.022**	0.205 (0.053–0.792)	**.022**
Fluconazole resistance absent (R)	1.826 (0.471–7.080)	.384	1.892 (0.407–8.786)	.416
Surgical treatment absent (R)	4.724 (1.5–14.872)	**.008**	5.764 (1.360–24.434)	**.017**

R, Reference; OR, odds ratio; aOR, adjusted odds ratio; CI, confidence interval.

## Discussion

This study presents a comprehensive analysis of all accessible osteoarticular *Candida* infections in Turkey during the past decade. The analysis revealed species distribution and clinical features that differ in some aspects from those described in the literature.

Gamaletsou et al. reported a *C. albicans* isolation proportion of 63% in a series of *Candida* arthritis cases involving 112 paediatric and adult patients. The same author reported a proportion of 65% in a series of 207 cases of *Candida* osteomyelitis.^11,12^ Among vertebral *Candida* infections, *C. albicans* was the most frequently isolated species, reported in 56.1% of cases in a study by Candura et al. and in 75% of cases in a series by Richaud et al.^13,14^ The prevalence of non-*albicans Candida* spp. in our study, which just exceeded 50%, highlights a striking epidemiological shift consistent with the global trend observed in invasive *Candida* infections.^15^ Among the non-*albicans Candida* spp., *C. parapsilosis* was the most common, detected in 15 cases. Notably, *C. auris*, an emerging pathogen of global public health concern, was also identified.^16^ Fluconazole resistance was low among *C. albicans* isolates (3%; 1/36), whereas it was high among non-*albicans* yeasts (27%; 10/37). According to the findings of a study conducted by Díaz-García et al. in Spain, there is an increase in fluconazole resistance in *Candida* spp. from 6.8% in 2019 to 16% in 2022. This increase was largely attributed to *C. parapsilosis*, for which the resistance rate increased dramatically, from 2.6% to 36.6%, over the same period. Consistent with this trend, in our study, the most frequently isolated non-*albicans* species was *C. parapsilosis*, which may partly explain the relatively high fluconazole resistance observed among non-*albicans* yeasts.^17^

An analysis of the affected anatomical regions revealed that the vertebrae and intervertebral discs, phalangeal bones in the hands and feet, and knee joints were the most commonly affected areas, which is consistent with the literature.^12,11^

It has been documented that bacterial co-infections can exacerbate *Candida* infections.^18^ In particular, bacterial co-infection has been associated with a poor prognosis in fungal prosthetic infections.^19^ In our study, co-infection was detected in slightly more than half of the patients (52.1%; 38/73), predominantly Gram-positive bacteria such as coagulase-negative *Staphylococcus* and *Enterococcus* species. However, the presence of bacterial co-infection did not significantly affect recovery (*P* = .911).

When comorbidities and predisposing factors were evaluated, the history of major surgery and broad-spectrum antibiotic use were identified and consistent with the literature. However, in our case series, diabetes, particularly insulin-dependent diabetes, emerged as a prominent factor. While the literature often reports osteoarticular infections as secondary to candidaemia, in our study, these infections were more frequently associated with trauma or surgical intervention involving the affected joint or bone.^11,12^

In terms of clinical findings, pain, limited joint mobility, oedema, and increased warmth were observed, consistent with previous reports. However, laboratory findings showed that the mean leukocyte count at diagnosis was 8901/µl, median CRP level was 31 mg/l, and mean erythrocyte sedimentation rate was 54.18 mm/h. These values were lower than those reported in previous studies. This may be explained by the higher median age of our study population (65 years). Most of our patients had access to modern radiological imaging modalities, such as CT or MRI, and radiological detection of osteomyelitis was achieved in 51 of 73 patients (69.9%). This is consistent with the findings reported in the literature. However, nuclear medicine imaging techniques were utilised to a lesser extent than previously reported.^11,12^

According to the 2016 Infectious Diseases Society of America candidiasis guidelines, the advised initial treatment for *Candida* osteomyelitis is fluconazole, with a treatment duration ranging from 6 to 12 months. For septic arthritis, the recommended treatment duration is approximately 6 weeks, although this can be extended to 3–6 months if joint prostheses are present. Fluconazole is the recommended first-line treatment for both cases. Surgical debridement is strongly recommended for osteomyelitis and surgical drainage for arthritis.^20^ In 2025, the European Confederation of Medical Mycology, the International Society for Human and Animal Mycology, and the American Society for Microbiology collaborated to publish an updated global guideline on the diagnosis and treatment of candidiasis. According to the latest recommendations, local epidemiological data and antifungal susceptibility test results should be considered in the management of osteoarticular *Candida* infections. Following surgical debridement, initial treatment with echinocandins or liposomal amphotericin B was advised, followed by oral fluconazole for 6–12 months. The treatment duration may be individualised based on clinical conditions. If fluconazole susceptibility is confirmed, therapy can be initiated directly with fluconazole and continued as appropriate.^21^ In a review of 211 cases of *Candida* osteomyelitis by Slenker et al., treatment was often initiated with amphotericin B.^22^ In our study, 54 patients received fluconazole, 29 received an echinocandin, and 6 received liposomal amphotericin B. Treatment regimens were modified based on the susceptibility results. The median treatment duration was 90 days, and the median follow-up duration was 180 days. Miller et al. reported that a median treatment duration of 45 days with aggressive surgical debridement would be sufficient for recovery from *Candida* osteoarticular infections.^23^ Similarly, in our study, multivariate analysis of the factors influencing clinical recovery highlighted the importance of surgical intervention (adjusted odds ratio [aOR], 5.764; 95% confidence interval [CI], 1.360–24.434; *P* = .017). Fluconazole resistance, *Candida* species, and treatment duration were not found to be statistically significant.

Another significant factor influencing total recovery in multivariate analysis was the presence of diabetes mellitus (aOR, 0.205; 95% CI, 0.053–0.792; *P* = .022). Globally, 463 million adults were living with diabetes in 2019 (9.3%), and this number is projected to increase to 578 million (10.2%) by 2030. The incidence of diabetes is increasing, especially in low- and middle-income countries, and it is considered an important predisposing factor for *Candida* infections owing to its immunosuppressive effects.^24,25^

The most significant limitations of our study are its retrospective design and the heterogeneity of the microbiological analysis methods employed by different contributing centres. In addition, the follow-up period was limited, varied between patients, and was generally shorter in those with partial recovery, which may have affected the detection of late recurrences. The study was strengthened by the large sample size and involvement of centres from different regions of Turkey. This was coordinated by the Turkish Society of Clinical Microbiology and Infectious Diseases.

In conclusion, osteoarticular *Candida* infections are challenging to treat and often require prolonged therapy. This multicentre study provides epidemiological data and fluconazole resistance rates of *Candida* species causing osteoarticular infections in Turkey, highlights the occurrence of *C. auris* in this cohort, and identifies surgical intervention and diabetes mellitus as factors significantly associated with recovery.

## Data Availability

Data will be shared via e-mail upon request.
